# Yolk-shelled silver nanowire@amorphous metal-organic framework for controlled drug delivery and light-promoting infected wound healing

**DOI:** 10.1093/rb/rbae056

**Published:** 2024-05-22

**Authors:** Yueyan Yang, Xu Sun, Shengyan Wang, Zhe Tang, Siyuan Luo, Jianjun Shi, Xiaolu Zhuo, Jinjin Zhu, Han Zhang, Xiangdong Kong

**Affiliations:** Institute for Smart Biomedical Materials, School of Materials Science & Engineering, Zhejiang Sci-Tech University, Hangzhou 310000, PR China; Zhejiang-Mauritius Joint Research Center for Biomaterials and Tissue Engineering, Zhejiang Sci-Tech University, Hangzhou 310018, PR China; Institute for Smart Biomedical Materials, School of Materials Science & Engineering, Zhejiang Sci-Tech University, Hangzhou 310000, PR China; Zhejiang-Mauritius Joint Research Center for Biomaterials and Tissue Engineering, Zhejiang Sci-Tech University, Hangzhou 310018, PR China; School of Science Engineering, The Chinese University of Hong Kong, Shenzhen, Guangdong 518172, PR China; Institute for Smart Biomedical Materials, School of Materials Science & Engineering, Zhejiang Sci-Tech University, Hangzhou 310000, PR China; Zhejiang-Mauritius Joint Research Center for Biomaterials and Tissue Engineering, Zhejiang Sci-Tech University, Hangzhou 310018, PR China; Institute for Smart Biomedical Materials, School of Materials Science & Engineering, Zhejiang Sci-Tech University, Hangzhou 310000, PR China; Zhejiang-Mauritius Joint Research Center for Biomaterials and Tissue Engineering, Zhejiang Sci-Tech University, Hangzhou 310018, PR China; Institute for Smart Biomedical Materials, School of Materials Science & Engineering, Zhejiang Sci-Tech University, Hangzhou 310000, PR China; Zhejiang-Mauritius Joint Research Center for Biomaterials and Tissue Engineering, Zhejiang Sci-Tech University, Hangzhou 310018, PR China; School of Science Engineering, The Chinese University of Hong Kong, Shenzhen, Guangdong 518172, PR China; Department of Orthopaedic Surgery, Sir Run Run Shaw Hospital, Zhejiang University School of Medicine & Key Laboratory of Musculoskeletal System Degeneration and Regeneration Translational Research of Zhejiang, Hangzhou 310016, PR China; Institute for Smart Biomedical Materials, School of Materials Science & Engineering, Zhejiang Sci-Tech University, Hangzhou 310000, PR China; Zhejiang-Mauritius Joint Research Center for Biomaterials and Tissue Engineering, Zhejiang Sci-Tech University, Hangzhou 310018, PR China; Institute for Smart Biomedical Materials, School of Materials Science & Engineering, Zhejiang Sci-Tech University, Hangzhou 310000, PR China; Zhejiang-Mauritius Joint Research Center for Biomaterials and Tissue Engineering, Zhejiang Sci-Tech University, Hangzhou 310018, PR China

**Keywords:** plasmonic nanocomposites, multifunctionality, amorphous metal–organic framework, controlled drug delivery, infected wound healing

## Abstract

Bacteria-infected wounds healing has been greatly hindered by antibiotic resistance and persistent inflammation. It is crucial to develop multifunctional nanocomposites that possess effective antibacterial properties and can simultaneously accelerate the wound healing process to overcome the above challenges. Herein, we prepared a yolk–shell structured Ag nanowires (NWs)@amorphous hollow ZIF-67 by etching ZIF-67 onto the Ag NWs for infected wound healing for the first time. The etched hollow structure of amorphous ZIF-67 in the nanocomposite makes it a promising platform for loading healing-promoting drugs. We extensively studied the antibacterial and healing-promoting properties of the curcumin (CCM)-loaded nanocomposite (Ag NWs@C-HZ67). Ag NWs, being noble metal materials with plasmonic effects, can absorb a broad range of natural light and convert it to thermal energy. This photothermal conversion further improves the release of antibacterial components and wound healing drugs when exposed to light. During the healing process of an infected wound, Ag and Co ions were released from Ag NWs@C-HZ67 upon direct contact with the wound exudate and under the influence of light irradiation. Simultaneously, the loaded CCM leaked out to repair the infected wound. The minimum inhibitory concentrations of the Ag NWs@C-HZ67 groups against *Escherichia coli* and *Staphylococcus aureus* bacteria decreased to 3 and 3 μg ml^−1^ when exposed to white light. Furthermore, an *in vivo* assessment of infected wound healing demonstrated that combining Ag NWs@C-HZ67 with light significantly accelerated the wound healing process, achieving 70% healing by the 6th day and almost complete healing by the 8th day. This advanced nanocomposite, consisting of components that possess antibacterial and growth-promoting properties, offers a safe, effective and clinically-translatable solution for accelerating the healing process of infected wounds.

## Graphical Abstract

**Figure:**
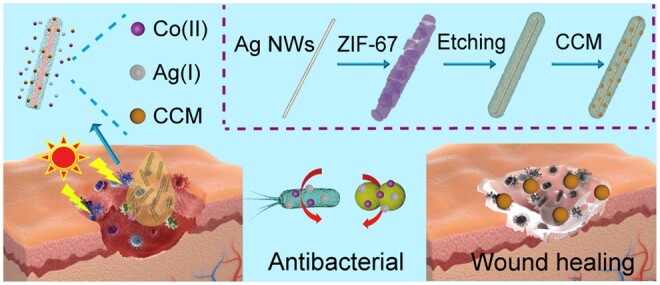

## Introduction

Bacteria, being pathogenic microorganisms, are responsible for causing infectious diseases that persistently afflict millions of individuals globally each year, often leading to high fatality rates [1]. Antibiotics have been commonly employed in the treatment of bacterial infections due to their broad-spectrum antimicrobial effects, significantly reducing the incidence and mortality rates caused by infections. Nevertheless, the excessive utilization of antibiotics has resulted in an inevitable surge in the variety and abundance of antibiotic-resistant strains [2, 3]. On November 21, 2023, the World Health Organization issued a latest report on antimicrobial resistance (AMR), revealing that AMR has emerged as a leading global challenge in the fields of public health and development. It is estimated that bacterial AMR directly caused 1.27 million deaths worldwide in 2019 and played a contributing role in 4.95 million fatalities [4]. Therefore, researchers in this field should actively develop new antibiotics, antibiotic delivery systems or new broad-spectrum antimicrobial agents as alternatives to alleviate the problem of AMR in bacteria and increase human capacity to combat resistant bacteria.

In the field of tissue engineering restoration, AMR and persistent inflammation pose significant obstacles in the treatment of bacterial-infected wounds [5]. The need for alternative approaches to combat AMR has motivated significant interest in the field of nanomaterials as potential nano-antimicrobials [6–8]. Among them, multifunctional nanocomposites integrating the advantages of two or more base materials have attracted great attention owing to their synergistic effects and enhanced properties when compared to the individual base counterparts [9, 10]. The nanocomposite encompasses a diverse category of materials, ranging from 3D and 2D composites to 1D NWs and 0D core shells [11–13]. The properties of nanocomposites are influenced not only by the used individual components but also by their morphology and interface characteristics. The enhanced properties exhibited by nanocomposites compared to their constituent materials endow them abilities for a broad range of energy, environmental and biomedical applications [14, 15].

Extensive effort has been focused on noble metal-based multifunctional nanomaterials, which involve the combination of metal nanoparticles (NPs) with various inorganic/organic nanocomponents to achieve the desired multifunctional properties. It is because that at nanoscale, noble metal nanomaterials display different chemical, physical and biological properties compared to their bulk counterparts due to the plasmonic effects [16]. Plasmonic metal NPs can effectively enhance light–matter interactions through sustaining coherent oscillations of surface charge density. These oscillations, known as localized surface plasmon resonance, can be finely adjusted to provide large optical cross-sections even beyond the spectral range of molecular electronic absorptions [17]. For example, Ag and Au NPs possess a remarkable capacity to efficiently absorb light with adjustable wavelengths across a wide range of electromagnetic spectrum from ultraviolet (UV) to infrared. This tunability depends on the composition, shape and size of the NPs [18, 19]. Photoexcitation of localized surface plasmon resonances can lead to a series of photophysical responses, including strong local electric field enhancement [20], hot carriers generation [21] and photothermal conversion [22]. These optical properties of plasmonics drive a variety of antimicrobial effects based on plasmonic photocatalysis and/or photothermal therapy [23]. Ag NPs have been used as effective antimicrobial agents since the 19th century due to their broad-spectrum activity and nonresistance properties. Meanwhile, as a plasmonic nanomaterial, Ag NPs also exhibit localized surface plasmon resonance effects. These characteristics make Ag nanomaterials a rapidly developing novel light-based antimicrobial agent [24].

Metal–organic frameworks (MOFs) are a kind of low-density crystalline porous materials composed of metal nodes and organic linkers [25]. In recent years, MOF materials have become a promising antimicrobial material, and their excellent antimicrobial properties mainly depend on physical contact, organic ligands and metal ions, photothermal effects, oxidative stress and synergistic effects [26]. The large specific surface area, high porosity, good biodegradability and ease of functionalization of MOF materials further widen their application prospects in the field of antibacterial therapies [26]. Zeolitic imidazolate framework-67 (ZIF-67) has been found to inhibit the growth of bacteria. At sub-inhibitory levels, ZIF-67 can effectively diminish the biomass of preexisting biofilms formed by these pathogenic bacteria. In addition, ZIF-67 NPs exhibit negligible or minimal toxicity in mammalian cells at those concentrations possessing antibiofilm or antibacterial properties [27]. Furthermore, amorphous MOFs that lack long-range periodic orders but maintain the basic components and connection of their crystalline framework have recently emerged in the fields involve the collapse of porous MOF structures for the release of guest species [28]. Amorphous MOFs possess higher storage capacities and are more easily to be decomposed in aqueous solution compared to their crystalline counterparts. Therefore, the preparation of amorphous ZIF-67 will further enhance its drug delivery capacity and antibacterial efficiency.

**Figure 1.**
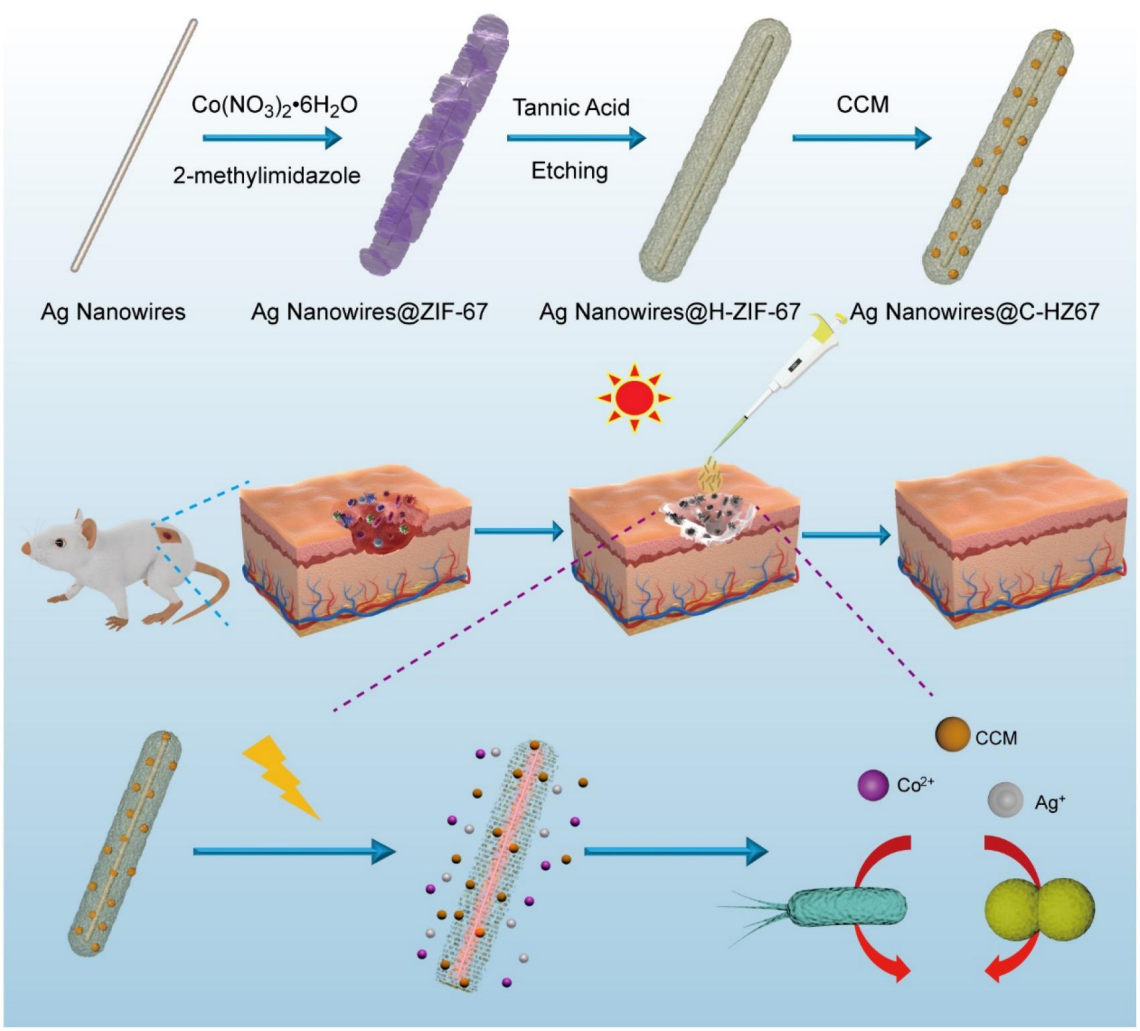
Schematic illustrating the synthesis procedure of Ag NWs@C-HZ67 for controlled drug delivery and infected wound healing.

In this study, we firstly synthesized ultra-long Ag NWs and deposited ZIF-67 composed of cobalt ions and imidazole on it. Then the yolk-shelled Ag NWs@amorphous hollow ZIF-67 is constructed by *in situ* etching of ZIF-67 on Ag NWs with tannic acid (TA). The hollow structure of the etched ZIF-67 in the nanocomposite allows it to serve as a superior drug delivery platform, loading effective antibacterial drugs and growth-promotion components such as CCM, giving the nanocomposite multifunctionality (Figure 1). The antibacterial and growth-promoting behaviors of the drug loaded nanocomposite (Ag NWs@C-HZ67) were then comprehensively studied. Ag NWs, as a noble metal material with plasmonic effects, can absorb a broad spectrum of natural light when their length reaches micrometer levels, further promoting the release of antibacterial components and wound healing drug under sunlight exposure. During the process of infected wound repairing, Ag and Co ions were released from the Ag NWs and amorphous hollow ZIF-67 by physical contact with the wound exudate under the stimulation of the light irradiation. The loaded drug was simultaneously leaked out from ZIF-67 to repair the infected wound. Conclusively, the minimal inhibitory concentration of the Ag NWs@C-HZ67 groups against *Escherichia coli* and *Staphylococcus aureus* bacteria under the white light irradiation is decreased to 3 and 3 μg ml^−1^, respectively. *In vivo* assessment of infected wound healing further demonstrated that the application of Ag NWs@C-HZ67 can greatly expedite the healing process under the light irradiation. By the 6th day, 70% of the wound had already healed, and nearly complete healing was achieved by the 8th day. This study introduces a promising antibacterial alternation that utilizes multifunctional nanocomposites comprising plasmonics and framework materials for the effective *in vivo* treatment of bacteria-infected wounds.

## Materials and methods

### Chemicals

TA (99%), 2-methylimidazole (2-MIM) (98%) and cobalt (II) nitrate hexahydrate (99%), agar powder, gel (strength ≥1200 g cm^−2^) were purchased from Shanghai Titan Technology Co., Ltd (Shanghai, China). Hexadecyltrimethylammonium bromide (CTAB, 99%), n-hexadecyltrimethylammonium chloride (CTAC, 99%) and AgNO_3_ (99%) were purchased from Sinopharm Group Chemical Reagent Co., Ltd (Shanghai, China). Citric acid (99%), L-ascorbic acid (AA, 99%), HAuCl_4_·3H_2_O (99.9%, trace metal base) were purchased from Merck (Shanghai, China). Polyvinylpyrrolidone (PVP, Mw was ∼24 000 Da), ethane glycol (99%) and yeast extract were purchased from Shanghai Yi En Chemical Technology Co., Ltd (Shanghai, China). Curcumin (CCM, 97%) was purchased from J&K SCIENTIFIC (Beijing, China). Dulbecco’s modified Eagle’s medium (DMEM) and fetal bovine serum (FBS) were purchased from Hyclone (Thermo Fisher, USA). PBS was purchased from Beijing Lanjieke Technology Co., Ltd. Calcein/PI cell activity cytotoxicity detection, EDTA solution, the methylthiazolyl tetrazolium (MTT) stain and penicillin streptomycin solution were purchased from Beyotime Biological Reagent Co., Ltd. Oxoid Tryptone was purchased from Thermo Fisher Scientific.

### Synthesis of Ag nanowires@hollow-ZIF-67

The Ag NWs samples were prepared by a seed-mediated method. Briefly, Au nanobipyramids (NBPs) were synthesized according to the reported method [29]. About 10.0 ml of Au NBPs solution (with the resonance peak wavelength at 800 nm and an optical density of 10) was centrifuged and redispersed into 120 ml of 0.08 M CTAC aqueous solution, then 20.0 ml of 0.01 M AgNO_3_ and 15 ml of 0.1 M AA aqueous solution were added to the mixed solution and stirred at 65°C for 4 h. After the reaction, the solution was left overnight to obtain Ag nanorods (NRs). Repeating the above steps, Ag NWs with diameters of 47 ± 2 nm and lengths of 4647 ± 239 nm were synthesized by using short Ag NRs as the seeds. About 0.5 g of PVP was added to 34.0 ml of ethylene glycol. Ag NWs were redispersed in the above solution and then sonicated for 10 min. Then, Ag NWs–PVP were centrifuged at 5000 rpm for 5 min.

The Ag NWs@ZIF-67 were prepared using the method reported before [30]. First, the obtained Ag NWs-PVP and 8.0 mmol of 2-methylimidazole were added in 40.0 ml of methanol under sonication for 30 min. After obtaining a homogeneous mixture, 2.0 mmol of Co(NO_3_)_2_·6H_2_O was dissolved in methanol (40.0 ml), and then quickly added to the former mixture under stirring. The resultant mixture was kept stirring for 1 h. The Ag NWs@ZIF-67 precipitate was obtained by centrifugation at 2000 rpm for 5 min. Then the precipitate was redispersed in 40.0 ml of absolute ethanol. About 75.0 mg of TA was dissolved in a mixture of 300 ml of ethanol and water (*V*_ethanol_:*V*_water_ = 1:1). Subsequently, 40.0 ml of Ag NWs@ZIF-67 methanol solution was rapidly added into the TA etching solution and kept stirring at 30°C for 1, 5 and 10 min, respectively. Finally, the precipitate was obtained by centrifugation (2000 rpm, 5 min) [31].

### Finite-difference time-domain simulations

The finite-difference time-domain (FDTD) simulation of an Ag NW was performed using Ansys Lumerical’s FDTD Solutions 2019b R6. The Ag NW was modeled in two dimensions, assuming an infinitely long wire with a circular cross-section having a radius of 23 nm. A total-field-scattering-field source of unit amplitude was used to generate a plane wave spanning the wavelength range of 250–1100 nm. The electric field was polarized in the transverse direction of the NW. The refractive index of surrounding medium was set to either 1 (representing air) or 1.33 (representing water). The dielectric function of Ag was fitted from Palik’s data. A mesh size of 0.2 nm was employed to calculate the extinction spectra and electric field distribution contours.

### Photothermal effect of Ag nanowires

To examine the photothermal conversion behavior of Ag NWs, the Ag NWs aqueous solution with an optical density of 3 was illuminated by the Xenon (Xe) lamp with a light intensity of 0.5 W cm^−2^ until the temperature was stabilized. The temperature changes were recorded by an infrared thermal imaging camera.

### Curcumin loading

First, an absorption–concentration standard curve of CCM in methanol was made by UV–Visible (Vis) spectrophotometer at 420 nm. The concentration of loaded CCM was determined through comparing the absorbance with the absorption–concentration standard curve. CCM methanol solution (100, 175, 250, 325, 500, 700, 1000 μg ml^−1^, 1 ml) was mixed with Ag NWs@H-ZIF-67 methanol solution (700 μg ml^−1^, 1 ml) and kept shaking for 24 h. Centrifugation (2000 rpm, 5 min) was carried out to collect the supernatant and test its absorption at 420 nm to calculate the drug loading rate. Drug loading behavior was calculated using the following equations:

$$\%\mathrm{Encapsulation}\;\mathrm{efficiency}\;=\frac{m_{0}-m_{c}}{m_{0}}\times 100$$

$$\%\mathrm{CCM}\;\mathrm{loading}=\frac{m_{0}-m_{c}}{m_{0}-m_{c}+m_{a}}\times 100$$

where *m*_0_ and *m_c_* represent the mass of original CCM and residual CCM, and *m_a_* represents the mass of Ag NWs@H-ZIF-67, respectively. The test was repeated three times.

### Curcumin and ion release

The solution combining PBS and Tween 20 (0.5 v/v%) with a pH of 7.4 was utilized to investigate the drug release characteristics. First, absorption–concentration standard curve of CCM in PBS-tween 20 was made by UV–Vis spectrophotometer at 420 nm. To examine the release behavior of CCM, 1 ml of Ag NWs@C-HZ67 was centrifuged and redispersed in 1 ml of PBS and Tween 20, then transferred to dialysis bags (molecular weight: 1000) and immersed in 12 ml of PBS and Tween 20 under gently shaking at 37°C.

The inductive coupled plasma atomic emission spectrometry (Agilent 5110, ICP-OES, USA) was utilized to analyse the released Ag and Co ions, which was obtained from the collected solution from the Ag NWs@H-ZIF-67 sample.

### Antibacterial performance

The antibacterial efficacy of Ag NWs@C-HZ67 was evaluated using *S*. *aureus* and *E*. *coli* as the model bacteria. The control group consisted of PBS, while the test group contained Ag NWs, ZIF-67, Ag NWs@H-ZIF-67 and Ag NWs@C-HZ67. The samples with different concentrations were dispersed in PBS and added to 96-well plates. Bactericidal activity of samples was estimated by comparing numbers of colony-forming units (CFUs) from bacterial cultures. The bacterial suspension in Luria–Bertani (LB) medium (1 × 10^6^ CFU ml^−1^) was mixed with the samples, with or without the Xe lamp illumination at 0.3 W cm^−2^ for 20 min, incubated for 6 h, and then tested for OD_600_ turbidity on a microplate reader. Bacteria survival rate was calculated according to the following equation:

$$\%\;\mathrm{survival}=C/C_{0}\times 100$$

where *C* is the bacterial turbidity of the sample group and *C*_0_ is the bacterial turbidity of the control group.

The bactericidal activity of Ag NWs@C-HZ67 was also evaluated by plate-killing assays, samples (100 μL, 6 μg ml^−1^ for Ag) were added to 100 μL bacterial suspension (1 × 10^6^ CFU ml^−1^) with or without Xe lamp illumination at 0.3 W cm^−2^ for 20 min. The obtained mixtures were then incubated in a shaker at 37°C for 6 h. The solutions were subsequently diluted by a factor of 10^6^ with PBS. Finally, the diluted solutions were spread onto agar plates and incubated at 37°C for a period of 24 h.

The bacterial damage was assessed through scanning electron microscopy (SEM) to observe any morphological changes between the treated and untreated strains. Samples (2 ml, 30 μg ml^−1^ of Ag) were introduced into a shaker at 37°C for 6 h, along with a bacterial suspension (1 × 10^6^ CFU ml^−1^). For SEM examination, all bacteria were fixed in a solution of 2.5% glutaraldehyde for a duration of 4 h and subsequently rinsed three times with PBS. Subsequently, the samples were redispersed in an ethanol solution, deposited onto a silicon wafer and air-dried for a period of 12 h. Finally, the samples were coated with a layer of Au conductive material to facilitate the observation of bacterial morphology by SEM.

### *In vitro* cytotoxicity

To evaluate the cytotoxicity of Ag NWs@C-HZ67 and Ag NWs@H-ZIF-67, mouse embryonic fibroblasts L929 and human umbilical vein endothelial cells (HUVEC) (1 × 10^4^ per well, 96-well plate) were incubated with different concentrations of Ag NWs@C-HZ67 and Ag NWs@H-ZIF-67 for 12 h under 5% CO_2_ at 37°C. Then the cell viability was measured by MTT assay.

To verify the growth-promoting performance of Ag NWs@C-HZ67, L929 cells were cultured in 6-well plates at a concentration of 2 × 10^5^ ml^−1^ and incubated at 37°C with 5% CO_2_ until a confluent monolayer was formed. Then, a monolayer of cells was scraped in a straight line using a sterile pipette tip (200 μL) and the cell debris was removed through PBS washing. Subsequently, 2.0 ml of the samples (3 μg ml^−1^) was added into the above plates. Following an incubation period of 12 h at 37°C and under 5% CO_2_ conditions, the migration of cells was observed using a microscope and analysed by the Image J software.

### *In vivo* wound healing evaluation

To assess the improved impact of the materials on infected wound healing, a mouse with *S.aureus* infected skin wound was modeled. All animal experiments in this work comply with the ‘Regulations on the Administration of Experimental Animals’ issued by the National Science and Technology Commission. The animal protocol was approved by the Animal Ethics Committee of Zhejiang Sci-Tech University (protocol number 20230930-01) and in accordance with the specifications of Care and Use of Laboratory Animals of Zhejiang Sci-Tech University. In detail, BALB/C mice (6–8 weeks) were first anesthetized by inhalation of isoflurane (1.5%). Then, the hair on the back of each mouse was removed, and a circular mark with a diameter of 0.8 cm was made using a hole punch in the depilated region. Subsequently, a full-thickness wound was created at the marked region using a medical scissor. Afterwards, 50.0 µL of *S.aureus* suspension (10^6^ CFU ml^−1^) was uniformly applied to each wound. After 24 h, 50 μL of sample solution was dropped onto each wound, and the light-treatment group was illuminated by Xe lamp at 0.3 W cm^−2^ for 20 min. The mice were sacrificed on day 9 for the routine blood test and histological examination. The wound healing results were observed by digital camera and analysed by Image J software.

### Instrumentations

Xe lamp (Beijing Zhongjiao Jinyuan Technology Co., Ltd, CEL-HXF300), transmission electron microscopy (TEM, JEM-2100, JEOL Ltd), SEM (GeminiSEM500, Carl Zeiss AG), X-ray diffraction (XRD, Bruker Corporation, A8 Advance), inverted biological microscope (Leica Microsystems, DMi1), UV–Vis (Shanghai Mapada Instruments Co., Ltd), microplate detector (Thermo Fisher Scientific, Varioskan LUX), confocal laser scanning microscope (Olympus Corporation, FV1200).

### Statistical analysis

All experimental data are depicted using the mean ± standard deviation (SD). Statistically remarkable values were assessed using *t*-test. **P <* *0.01 and ***P <* *0.001 both represent the difference between data was statistically significant.

## Results and discussion

### Synthesis and characterizations of Ag nanowires@hollow-ZIF-67

**Figure 2.**
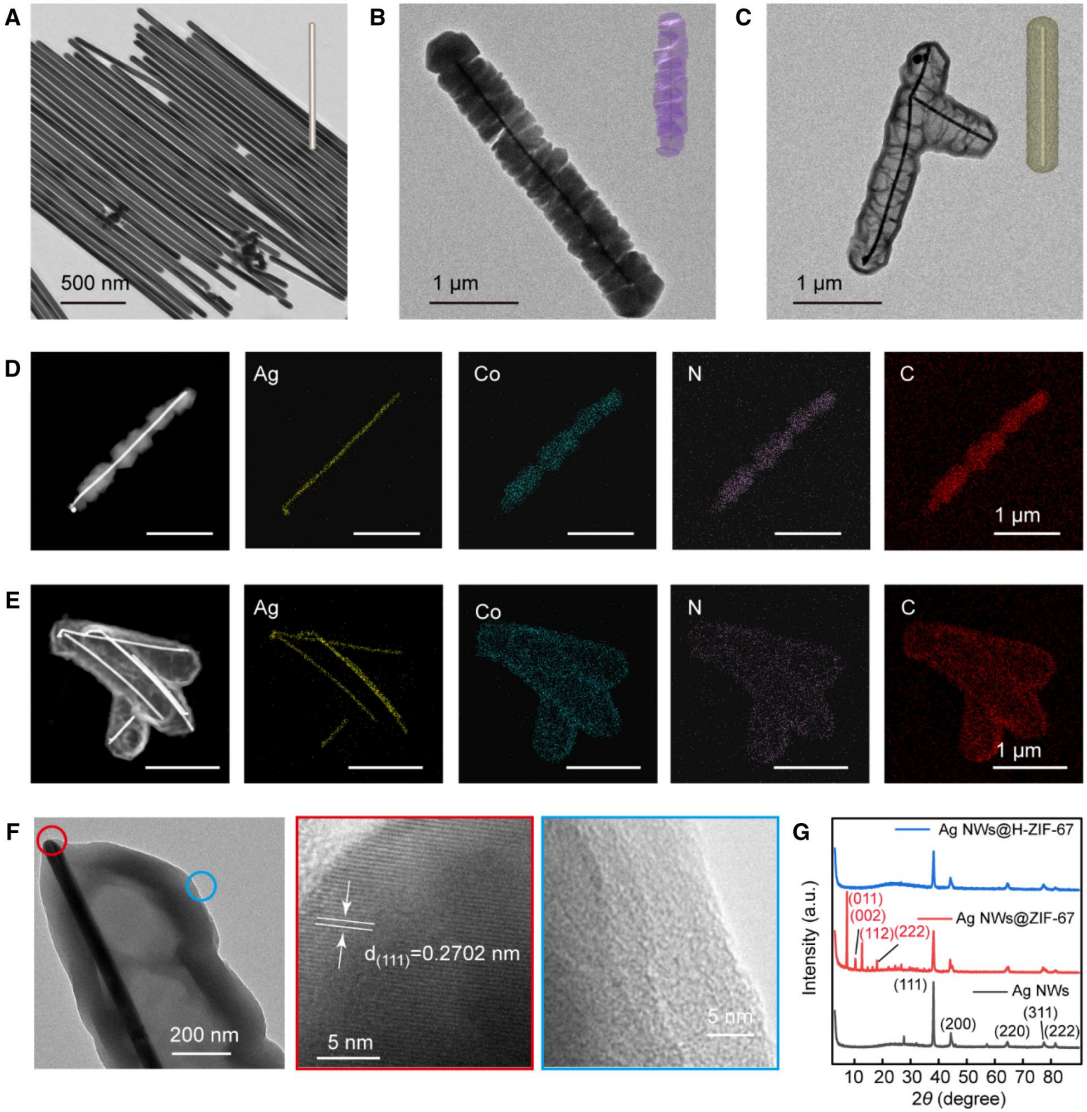
TEM images of Ag NWs (**A**), Ag NWs@ZIF-67 (**B**) and Ag NWs@H-ZIF-67 (**C**). TEM mapping images of Ag NWs@ZIF-67 (**D**) and Ag NWs@H-ZIF-67 (**E**). High resolution TEM (HRTEM) images of Ag NWs@H-ZIF-67 (**F**), and XRD spectrum of Ag NWs, Ag NWs@ZIF-67 and Ag NWs@H-ZIF-67 (**G**).

The structure diagram of the whole preparation process of Ag NWs@H-ZIF-67 is shown in Figure 1. The morphologies of Ag NWs, Ag NWs@ZIF-67 and etched Ag NWs@hollow-ZIF-67 (Ag NWs@H-ZIF-67) were observed by TEM (Figure 2A–C). As shown in Figure 2A, the micron-sized Ag NWs possess large aspect ratio with an average length of 4647 ± 239 nm and an average diameter of 47 ± 2 nm. It can be found that ZIF-67 components with irregular flocculent shapes adhere to the surface of Ag NWs, forming Ag NWs@ZIF-67 structure with an average thickness of 300 ± 11 nm (Figure 2B). After etching the prepared Ag NWs@ZIF-67 with TA solution, the ZIF-67 layer on the surface of Ag NWs becomes a hollow structure, Ag NWs@H-ZIF-67 were prepared (Figure 2C). The SEM images of the Ag NWs, Ag NWs@ZIF-67 and Ag NWs@H-ZIF-67 are presented in Figure S1. To reveal the composition of Ag NWs@ZIF-67 and Ag NWs@H-ZIF-67, the energy dispersive spectrometer (EDS) measurements and TEM elemental mapping were performed. Both Ag NWs@ZIF-67 and Ag NWs@H-ZIF-67 are composed of elements including Ag, Co, C and N (Figure 2D and E). The element’s content in Ag NWs@ZIF-67 is different from that in Ag NWs@H-ZIF-67, as shown in Figure S2. For Ag NWs@ZIF-67 sample, Ag and Co elements account for 80.57 and 19.43 wt%, respectively. For Ag NWs@H-ZIF-67 sample, Ag and Co elements account for 90.09 and 8.91 wt%, respectively. The content of Co decreased in the Ag NWs@H-ZIF-67 sample, indicating the hollowing of the inner structure of ZIF-67 on Ag NWs during the etching process.

To further investigate the crystalline structure of the samples, HRTEM analysis and XRD measurements were carried out (Figure 2F and G). The HRTEM image of the Ag NW component in Ag NWs@H-ZIF-67 exhibited obvious lattice fringes. The lattice fringe spacing was ∼0.270 nm, which matched with the (1 1 1) lattice plane of Ag element (red circle in Figure 2F) [32]. The HRTEM image of the hollow ZIF-67 site (the blue circle) indicated that a dense amorphous structure was formed. XRD measurements were performed to investigate the crystalline structures of Ag NWs, Ag NWs@ZIF-67 and Ag NWs@H-ZIF-67 composites (Figure 2G). The diffraction peaks at 38.6°, 44.7°, 64.9°, 77.7° and 81.8° can be well-indexed to the (1 1 1), (2 0 0), (2 2 0), (3 1 1) and (2 2 2) planes of the Ag (JCPDS No. 04-0783), respectively. The diffraction peaks at 7.3°, 10.4°, 12.7°, 14.7°, 16.5° and 17.9° can be well-indexed to the (0 1 1), (0 0 2), (1 1 2), (0 2 2), (0 1 3) and (2 2 2) planes of the ZIF-67, respectively [33]. Only Ag XRD patterns can be observed in Ag NWs@H-ZIF-67 sample, further demonstrating that the etched hollow ZIF-67 components on the surface of Ag NWs are amorphous. The etching process disrupted the crystalline structure of ZIF-67, leading to the amorphization of hollow ZIF-67. Due to the amorphous structure of hollow ZIF-67 shell, they exhibit a structural instability in the PBS phase (simulated physiological fluid). As shown in Figure S3, the structure of ZIF-67 and hollow ZIF-67 shell gradually collapsed and degraded after being stored in PBS solution for 12 h, especially the hollow ZIF-67 shell. However, both Ag NWs@ZIF-67 and Ag NWs@H-ZIF-67 are stable in methanol phase even for 30 days. This controllable stability of the hollow ZIF-67 actually makes Ag NWs@H-ZIF-67 composites a promising drug delivery vehicle for wound healing applications.

### Optical properties and photothermal effect

**Figure 3.**
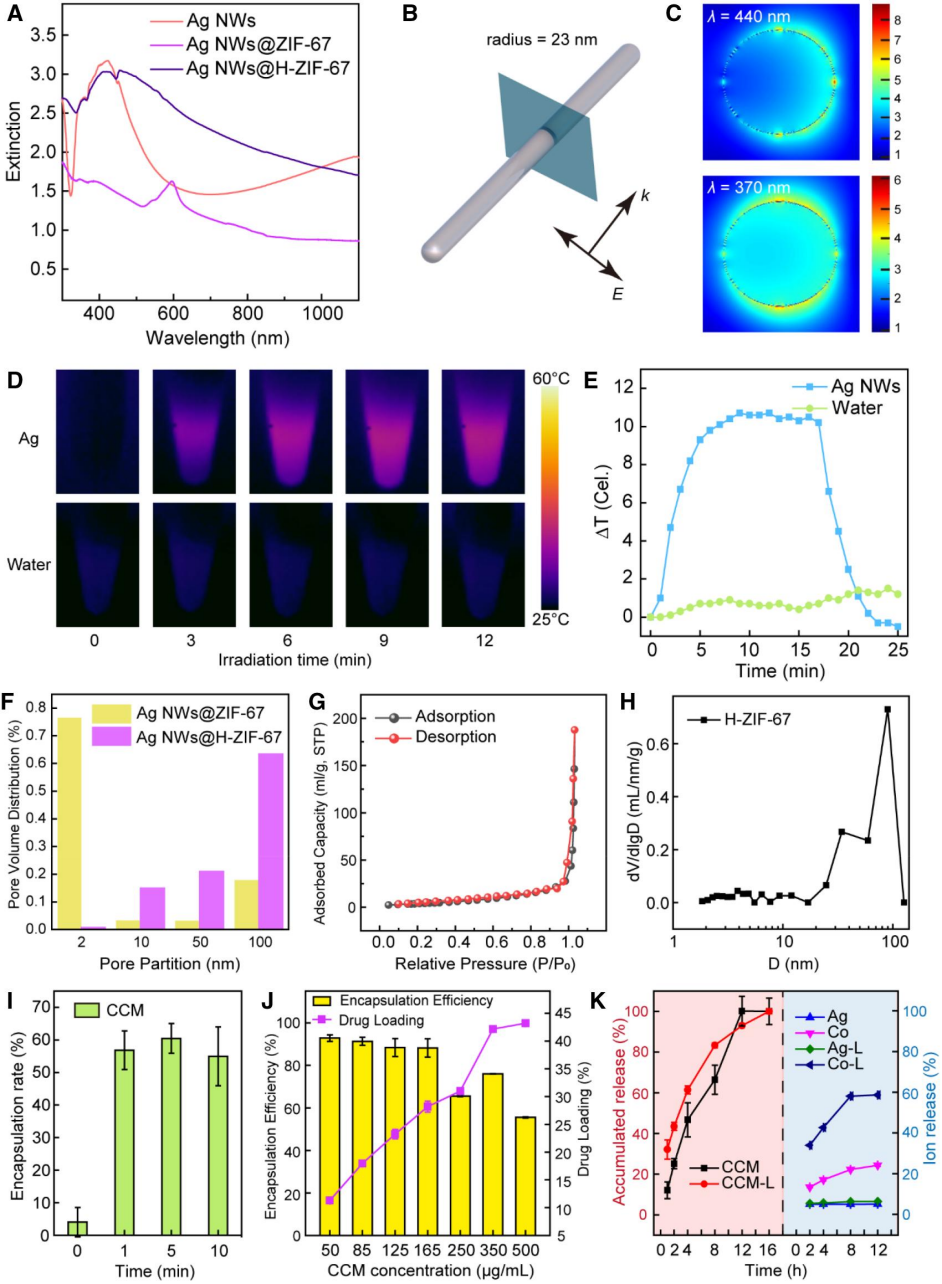
Extinction spectra of the Ag NWs, Ag NWs@ZIF-67 and Ag NWs@H-ZIF-67 (**A**). Electric field enhancement contours of a single Ag NW (**B**, **C**). The arrows in (B) show the incidence and polarization directions of the excitation light. Photothermal images (**D**) of the aqueous suspension of dispersed Ag NWs (OD = 3) under an irradiation of Xe lamp (0.5 W cm^−2^). Photothermal heating curves of aqueous suspensions of dispersed Ag NWs (**E**). Pore volume distribution of ZIF-67 and H-ZIF-67 (**F**). Isothermal adsorption–desorption curve of H-ZIF-67 (**G**). Pore size distribution curve of the H-ZIF-67 (**H**). The encapsulation rate of CCM in the Ag NWs@H-ZIF-67 (700 μg ml^−1^ of Ag) with different etching time periods (**I**). The encapsulation rate and drug loading of CCM in the Ag NWs@H-ZIF-67 with different concentrations of CCM (**J**). Drug and ion release values for the Ag NWs@C-HZ67 with and without the light irradiation (Xe lamp, 0.5 W cm^−2^) (**K**).

The physicochemical properties of Ag NWs, Ag NWs@ZIF-67 and Ag NWs@H-ZIF-67 were then carefully characterized. First, the UV–Vis extinction spectra of Ag NWs, Ag NWs@ZIF-67 and Ag NWs@H-ZIF-67 were tested to show their optical properties. Ag NWs have a peak extinction at 420 nm, meanwhile they possess the capability to absorb light across a broad spectrum, ranging from 300 to 1100 nm. After coating Ag NWs with ZIF-67 shell, the extinction peak of Ag NWs@ZIF-67 redshifted to around 590 nm. Ag NWs@H-ZIF-67 NPs also exhibit great extinction throughout a wide range of the light spectrum (300–1100 nm), with the peak extinction occurring at 450 nm (Figure 3A). When Ag NWs are exposed to a light, the incident photons can be absorbed and partially converted to heat. FDTD simulations can exhibit the intricate details of light–matter interactions, such as the distribution of electromagnetic near-field enhancement on Ag NWs [34]. Therefore, FDTD simulations were performed to visualize the light absorption and potential light-induced self-heating ability of Ag NWs (Figure 3B and C). The simulated Ag NW was modeled according to the measured geometrical parameters from TEM and SEM images. About 370 and 440 nm were chosen as the wavelength of incident light. The incident direction of light is set to be perpendicular to the cross-section of the Ag NW and parallel along the Ag NW (Figure 3B). We can find from the cross-sectional electric field enhancement contours of the Ag NW that the electric field enhancement occurred on the confined region near the surface of the Ag NW, demonstrating the surface confined light-induced electric field enhancement around the Ag NW (Figure 3C). The photothermal conversion properties of Ag NWs can be further verified by the photothermal heating images and curves (Figure 3D and E). Under the irradiation of a Xe lamp (0.5 W cm^−2^) for 9 min, the temperature of the Ag NWs solution increased by 10.9°C. In comparison, deionized water only exhibited a temperature increase of 0.8°C under the same condition. These findings demonstrate the considerable ability of Ag NWs to convert white light into thermal energy efficiently and rapidly. The photothermal–cooling cycling curves of Ag NWs, Ag NWs@H-ZIF-67 and Ag NWs@C-HZ67 demonstrated the excellent photothermal stability of the materials (Figure S4). Moreover, it is important to note that the temperature rise of Ag NWs might not be sufficient for directly eradicating bacteria, but can promote the decomposition of ZIF-67, subsequently leading to the release of ions and encapsulated drugs. This point of view will be elaborated on in the subsequent discussion.

### Controlled drug delivery and ion release behaviors

The porosity of H-ZIF-67 is a crucial parameter for drug loading and was assessed by the nitrogen adsorption–desorption tests at 77 K. The pore volume distributions of Ag MWs@ZIF-67 and Ag MWs@H-ZIF-67 samples revealed that the proportion of microporous structure of ZIF-67 gradually vanished while the proportion of large pores of H-ZIF-67 increased to 74.81% after the etching process (Figure 3F). The isothermal adsorption–desorption curves of H-ZIF-67 in Figure 3G exhibit the Type-III behavior, which is the characteristic of microporous materials [35]. The BET average pore diameter of ZIF-67 is 2.77 nm, while that of H-ZIF-67 is increased to 76.0316 nm (Figure 3H). The enlargement in pore size and volume after etching facilitates drug loading into the amorphous hollow ZIF-67. The drug loading and release behaviors of Ag NWs@H-ZIF-67 were examined by CCM, which is a drug simultaneously possessing antibacterial and tissue-healing properties. CCM can be loaded into MOF materials through the synergistic effect of chemical chelation and physical adsorption [36]. The CCM encapsulation efficiency can be adjusted by controlling the etching time periods during the preparation of Ag NWs@H-ZIF-67 (Figure 3I). The maximal CCM (250 μg ml^−1^) encapsulation efficiency reached 60% in the Ag NWs@H-ZIF-67 (700 μg ml^−1^ of Ag) sample with 5 min etching. The Ag NWs@ZIF-67 sample without etching process shows only ∼5% encapsulation efficiency. This illustrates that the hollow structure of ZIF-67 plays an important role in improving the encapsulation efficiency of drugs. At the same time, the etching time should be finely controlled, as excessive etching (for instance, to 10 min) may damage the surface structure of ZIF-67 and cause drug leakage. After determining the etching time, we further adjusted the CCM concentration to find out the optimal drug loading rate, which was calculated to be 43% in weight defined by the maximum absorption of CCM on 420 nm (Figure S5A, Figure 3J). The yolk–shell structured Ag NWs@H-ZIF-67 with large cavity offers the quite high drug loading capacity. The optimal CCM-loaded Ag NWs@H-ZIF-67 is named as Ag NWs@C-HZ67. Moreover, the release behaviors of CCM and ions were monitored in the solution of PBS and Tween 20 (0.5 v/v%) with a pH of 7.4 (physiological condition) treated with or without light irradiation (Figure S5B, Figure 3K). As seen in Figure 3H, the release performance of CCM and ions were greatly affected by the light irradiation. The release of CCM can reach nearly 100% at 16 h due to the destruction of the H-ZIF-67 shell in the PBS solution. The accumulated release rate of CCM in Ag NWs@C-HZ67 increases under the 0.5 W cm^−2^ light irradiation. Generally, the hollow ZIF-67 on Ag NWs will be decomposed to Co ions and 2-MIM fragments in aqueous solution, and then the Ag NWs can also be oxidized to release Ag^+^ [37]. Both the decomposition and oxidation are kinetic reactions, and thus the increase of temperature will accelerate the process and promote the release of Co and Ag ions. The ion release behaviors of the Ag NWs@C-HZ67 were also carefully investigated. The release amount of Ag^+^ was 4.4% without light irradiation, while a slight increase of Ag^+^ release (5.1%) can be observed after light irradiation. The light irradiation mainly promotes the release of Co ions. The release amount of Co ions increased from 22.3% to 59.8% at the incubation time of 12 h under the light irradiation, indicating that light-to-thermal conversion can promote the release of Co ions.

### *In vitro* antibacterial performance

**Figure 4.**
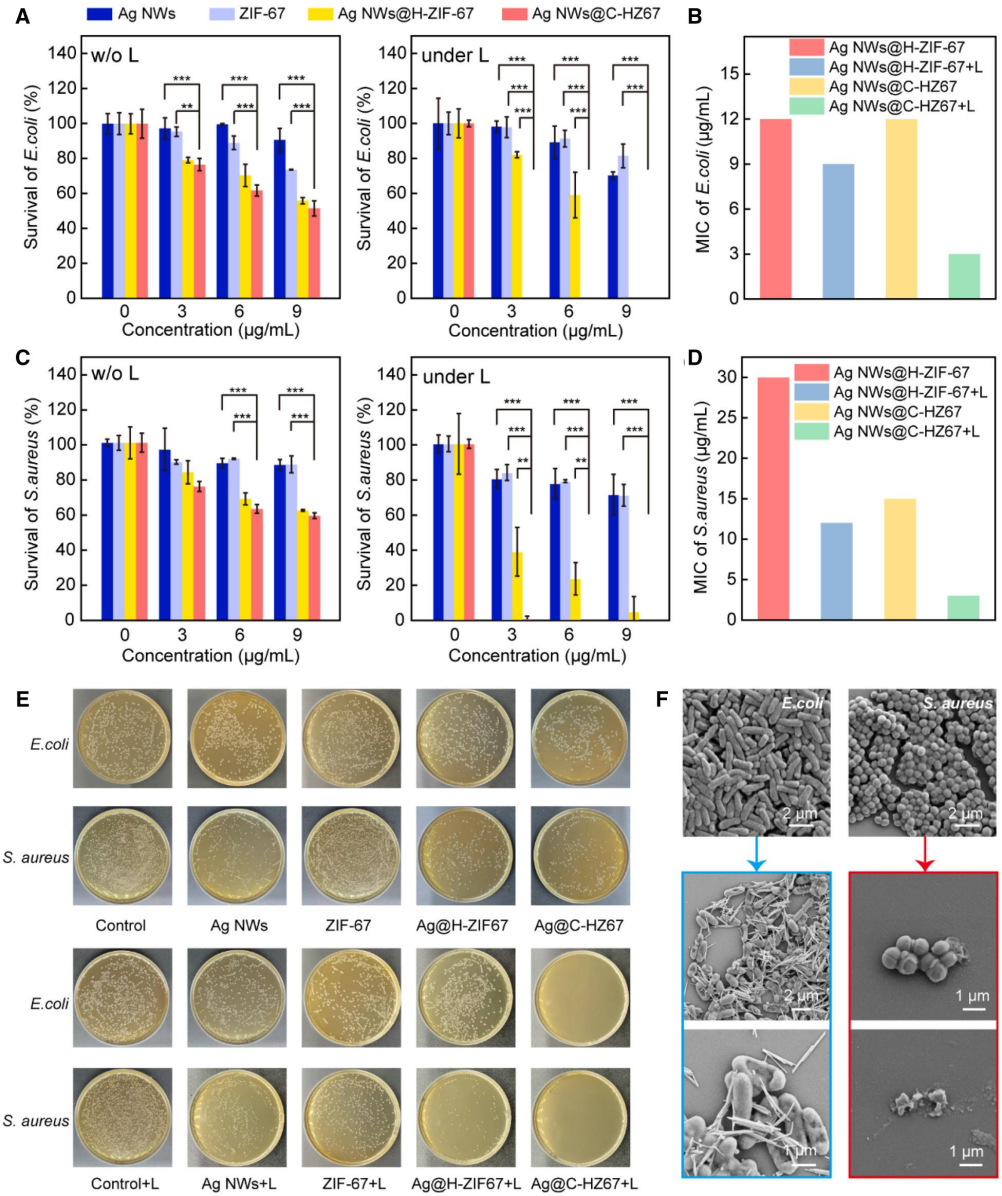
Determination of the antimicrobial turbidity of the materials against *E.coli* and *S.aureus* (**A**, **C**). MIC of Ag NWs@H-ZIF-67 and Ag NWs@C-HZ67 against *E.coli* and *S.aureus* with and without light irradiation (**B**, **D**). Photographs of survival bacteria clones on agar plates after different treatments (**E**). SEM images of *E.coli* and *S.aureus* after co-culturing with Ag NWs@C-HZ67 sample under light irradiation (**F**).

The *in vitro* antibacterial properties of Ag NWs, ZIF-67, Ag NWs@H-ZIF-67 and Ag NWs@C-HZ67 were assessed by measuring and comparing the absorbance at 600 nm of two bacterial models (*E.coli* and *S.aureus*) subjected to various treatments (Figure 4). Both Ag NWs and ZIF-67 groups cannot hinder the propagation of bacteria at a trace concentration of antibacterial agents with or without the light irradiation. Conversely, the Ag NWs@H-ZIF-67 and Ag NWs@C-HZ67 groups can effectively and efficiently eliminate two bacterial models upon exposure to white light (Figure 4A–D). The minimum inhibitory concentration (MIC) of the Ag NWs@H-ZIF-67 group against *E.coli* and *S.aureus* under the dark condition is 12 and 30 μg ml^−1^, respectively. While the MIC of the Ag NWs@C-HZ67 groups against *E.coli* and *S.aureus* under the dark condition is 12 and 15 μg ml^−1^, respectively. By loading the CCM components, the antibacterial activity of Ag NWs@C-HZ67 is better than that of Ag NWs@H-ZIF-67 against *S.aureus*. Furthermore, the difference in antimicrobial performance between the Ag NWs@H-ZIF-67 and Ag NWs@C-HZ67 group is more pronounced under the light irradiation of 0.3 W cm^−2^ for 20 min. The MIC of the Ag NWs@H-ZIF-67 group against *E.coli* and *S.aureus* under light irradiation is decreased to 9 and 12 μg ml^−1^ compared to the dark counterparts. While the MIC of the Ag NWs@C-HZ67 groups against *E.coli* and *S.aureus* under the light irradiation is decreased to 3 and 3 μg ml^−1^, respectively. The corresponding photographs of bacterial colonies exhibit a similar trend to the bacterial turbidity experiments (Figure 4E). There are no obvious antibacterial properties showed in the Ag NWs and ZIF-67 groups from the plate counting method. Compared to the Ag NWs@C-HZ67 group under the dark condition, the bacterial colonies of both *E.coli* and *S.aureus* were completely removed with the treatment of Ag NWs@C-HZ67 group under light irradiation, highlighting that the photothermal effect could largely enhance the antibacterial rate of the Ag NWs@C-HZ67 group to achieve an ideal sterilization effect within a short time.

### Bacterial morphology and antibacterial mechanism

The morphological changes of *E.coli* and *S.aureus* before and after the treatment with the Ag NWs@C-HZ67 sample under light irradiation were investigated by SEM images (Figure 4F). We can observe that only Ag NWs remain on the surface of bacteria after the light treatment, demonstrating the decomposition of the hollow ZIF-67 shells. The morphology of bacteria becomes wrinkle, deformation and localized dimple after the treatment, indicating that the as prepared Ag NWs@C-HZ67 NPs was able to effectively kill bacteria by damaging the cell walls and further causing the loss of cellular contents.

As an extremely efficient light-induced antimicrobial agent, the antibacterial mechanism of Ag NWs@C-HZ67 NPs mainly encompasses the following three aspects. First, under the light irradiation, 2-MIM anions and Co^2+^ released from the decomposition of hollow ZIF-67 shell can significantly inhibit the biomass of pre-established biofilms of pathogenic bacteria [27]. Second, a small amount of Ag ions released from Ag NWs can initiate oxidative stress within bacteria, leading to decreased activity of intracellular enzymes and ultimately resulting in bacterial death [38]. Third, during the degradation of H-ZIF-67 shells, the encapsulated CCM leaked out. CCM itself is a broad-spectrum antimicrobial agent [39]. The above factors make Ag NWs@C-HZ67 NPs an efficient bactericidal agent. Therefore, compared to the other nanomaterials comprising metal components and/or MOF in the previous reports (Table 1) [40–48], Ag NWs@C-HZ67 NPs in this work possess a remarkable antimicrobial efficacy against both *E.coli* and *S.aureus.*

**Table 1.** Comparative assessment of the antimicrobial efficacy of nanomaterials comprising plasmonic components and/or MOF materials

Material	Effective concentration (μg ml^−1^)	Refs.
Ag NP-modified 2D Zr-ferrocene-MOF nanosheets	*E.coli* 50 *S.aureus* 100	[40]
Chlorin e6 (Ce6)-loaded zinc-MOF	*S.aureus* 380	[41]
MOF(Fe–Cu)/GO_*x*_-polyacrylamide	*E.coli* 50 *S.aureus* 50	[42]
Cu-MOF@Ag NPs	*S.aureus* 20	[43]
Fe_3_O_4_/Ag nanospheres	*E.coli* 800 *S.aureus* 1000	[44]
Ag NPs	*S.aureus* 625	[45]
Au–Ag NPs	*E.coli* 25 *S.aureus* 30	[46]
Ag NPs@ACM-1	*E.coli* 39.1 *S.aureus* 39.1	[47]
Ag-decorated quercetin NPs	*E.coli* 15 *S.aureus* 15	[48]
Ag NWs@C-HZ67	*E.coli* 3 *S.aureus* 3	This work

### *In vitro* biocompatibility

**Figure 5.**
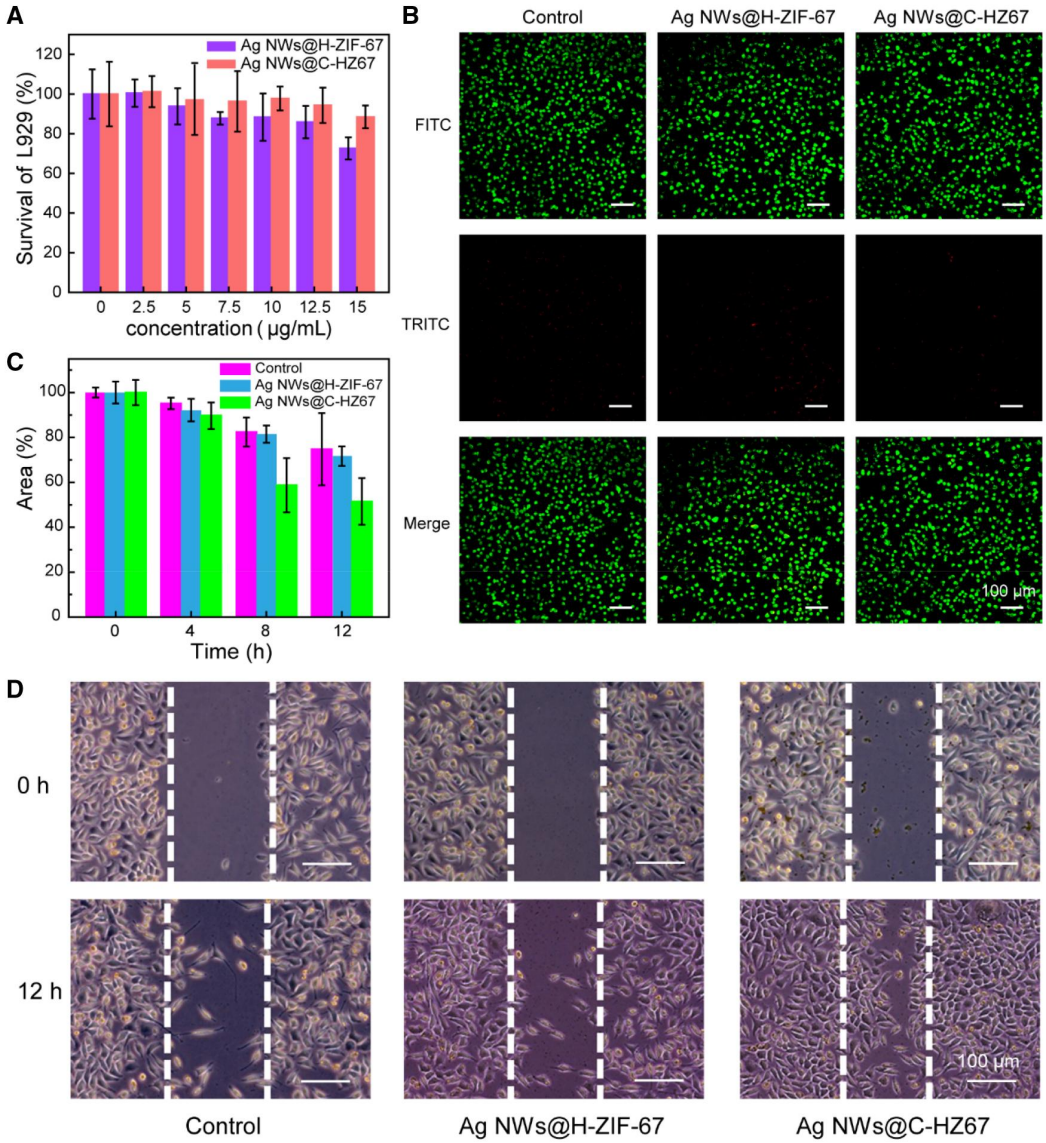
Cell viability of L929 cells incubated with Ag NWs@H-ZIF-67 and Ag NWs@C-HZ67 for 12 h (**A**). Live/dead staining of L929 cells incubated with samples after cell co-cultured for 12 h (**B**). Quantitative analysis of scratch area after 4, 8 and 12 h (**C**). Representative images of scratch method of L929 cells after 12 h (**D**).

HUVEC and mouse embryonic fibroblasts L929 cells were selected to evaluate the cytotoxicity of Ag NWs@H-ZIF-67 and Ag NWs@C-HZ67 (Figure 5A and Figure S6). After incubating with the materials at concentrations showing antibacterial activities, the cell viability of both cell types remained above 90% for an incubation duration of 12 h, demonstrating the superior biosafety of the materials. To further explore the cytotoxicity of the aforementioned two nanomaterials, live/dead cell assays were conducted on L929 cells. The results depicted in Figure 5B demonstrate that the majority of L929 cells displayed green fluorescence, indicating that they were live cells. This observation was consistent across the control group, as well as the Ag NWs@H-ZIF-67 and Ag NWs@C-HZ67 groups, exhibiting their excellent biosafety for use in biomedical applications.

### Accelerated *in vitro* cell migration

CCM, known for its bio-functional properties such as antioxidant, radical scavenging, antimicrobial and anti-inflammatory activities, has been extensively studied for several years [39]. These properties are essential in the wound healing process as they contribute significantly to its management. Additionally, CCM promotes the production of growth factors that play a vital role in healing wounds, thus expediting the process of wound restoration. The cell scratch assay was chosen to simulate the *in vitro* wound healing process to investigate the effect of CCM released from Ag NWs@C-HZ67 on the migration of L929 cells. As illustrated in Figure 5C and D and Figure S7, it was observed that the Ag NWs@C-HZ67 group exhibited the highest rate of cell migration compared to the other treatment groups. This suggests that the controlled release of CCM from Ag NWs@C-HZ67 can effectively promote cell migration, which facilitates the *in vivo* wound healing process.

### *In vivo* infected wound healing assessment

**Figure 6.**
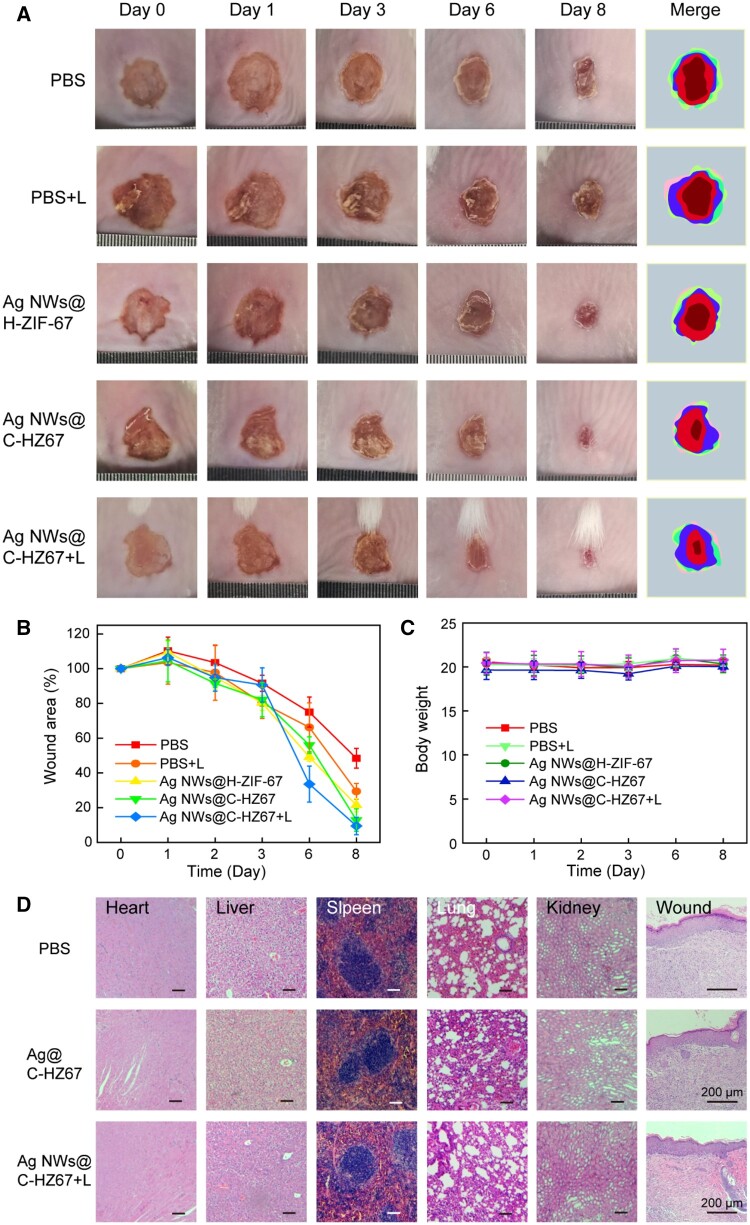
Pictures of wounds were taken at Day 0, 1, 3, 6 and 8 after healing with PBS, PBS with light, Ag NWs@H-ZIF-67, Ag NWs@C-HZ67 and Ag NWs@C-HZ67 with light, and their corresponding schematic representations of the wound healing processes (**A**). The quantitative wound area (**B**). The weight of mice during the course of treatment (**C**). Heart, liver, spleen, lung, kidney and wound of H&E staining photos of the control group, Ag NWs@C-HZ67 and Ag NWs@C-HZ67 + L group on the 9th day (**D**).

To investigate the healing ability of the prepared nanomaterials, an experimental model was created by establishing a full-thickness *S.aureus* infected skin defect on the back of mouse, resulting in a circular wound with a diameter of ∼0.8 cm. The mice were randomly divided into the PBS control group, the PBS with light (PBS + L) group, the Ag NWs@H-ZIF-67 group, the Ag NWs@C-HZ67 group and the Ag NWs@C-HZ67 with light (Ag NWs@C-HZ67 + L) group. The wound healing process were recorded and assessed at 0, 1, 3, 6 and 8 days, respectively (Figure 6A). The Ag NWs@C-HZ67 + L group exhibited an accelerated wound healing rate compared to the other groups. By the 6th day, the wounds in this group had already healed 70%, and by the 8th day, they were almost completely healed. However, the wounds of the PBS, PBS + L and Ag NWs@H-ZIF-67 groups showed slow healing progress, which could not wholly heal after 8 days. As shown in Figure 6B, quantitative analysis of wound area also confirmed the superiority of the Ag NWs@C-HZ67 + L group over the other groups. The enhanced healing rate of the Ag NWs@C-HZ67 + L could be attributed to the light-promoting release of Ag^+^, Co^2+^ and CCM for antibacterial wound treatment. Additionally, the bacterial contents in the wounds at days 1, 2 and 3 under the treatment of PBS, Ag NWs@C-HZ67 and Ag NWs@C-HZ67 + L groups were investigated, respectively (Figure S8). The results further demonstrated the superior antibacterial capacity of the Ag NWs@C-HZ67 and Ag NWs@C-HZ67 + L groups. The body weight of all treated mice was then monitored (Figure 6C) and no significant changes were observed, indicating our materials would not affect the growth and health of the mice. The complete blood routine analysis was further introduced to evaluate the biocompatibility of Ag NWs@H-ZIF-67 and Ag NWs@C-HZ67 nanomaterials *in vivo* (Figure S9). Compared with the PBS control group, there were no significant changes in the blood routine in the material groups. Furthermore, the biocompatibility and wound healing activity of the material groups were confirmed by H&E staining (Figure 6D and Figure S10). Mice major organs including the heart, lung, liver, kindly and spleen did not show any inflammation or damage after the treatment by materials for 9 days, further demonstrating the Ag NWs@H-ZIF-67 and Ag NWs@C-HZ67 nanomaterials are not toxic to the treated animals at the experimental dose. The observation of tissue samples from the PBS group revealed the presence of a broken epidermis. Meanwhile, the wound tissue obtained from mice in the PBS + L and Ag NWs@H-ZIF-67 groups displayed insufficient healing, characterized by the presence of abundant neutrophils. However, treatment with the Ag NWs@C-HZ67 + L group resulted in a complete re-epithelialization and significant reduction in inflammation while the wound sections in the Ag NWs@C-HZ67 + L treatment group exhibited the development of hair follicle, thus indicating a remarkable efficiency in wound healing (Figure 6D).

## Conclusion

In summary, we achieved a yolk-shelled Ag NWs@amorphous hollow ZIF-67 structure by etching ZIF-67 on the Ag NWs. The hollow structure of the etched ZIF-67 in the nanocomposite allows it to be an excellent platform for CCM loading. We conducted a comprehensive study on the antibacterial and wound healing properties of the CCM-loaded nanocomposite (Ag NWs@C-HZ67). Ag NWs, as a noble metal material with plasmonic effects, can absorb a wide range of natural light when they reach lengths in the micrometer range. This absorption of light further enhances the release of antibacterial components and wound healing drugs when exposed to sunlight. During the process of healing an infected wound, the Ag and Co ions are released from Ag NWs@C-HZ67 upon physical contact with the wound exudate and under the stimulation of light irradiation. At the same time, the loaded drug leaks out from the ZIF-67 to repair the infected wound. The MIC of the Ag NWs@C-HZ67 groups to inhibit the growth of *E.coli* and *S.aureus* bacteria decreases to 3 and 3 μg ml^−1^, respectively, under white light irradiation. Additionally, an *in vivo* assessment of infected wound healing further demonstrated that Ag NWs@C-HZ67 combined with light can significantly accelerate the wound healing process, with 70% healing achieved by the 6th day and almost complete healing by the 8th day. This work provides a new perspective for the design of multifunctional nanocomposites applicable in treating bacterial infections, which will be further studied in the future.

## Supplementary Material

rbae056_Supplementary_Data

## Data Availability

The datasets supporting the conclusions of this article are included within the article and its additional files.
