# Supplementary material for: Yolk-shelled silver nanowire@amorphous metal-organic framework for controlled drug delivery and light-promoting infected wound healing
Source: Regen Biomater. 2024 May 22;11:rbae056. doi: 10.1093/rb/rbae056 (PMC11153340; doi:10.1093/rb/rbae056)
Supplement: rbae056_Supplementary_Data [file rbae056_supplementary_data.docx]

**Supporting Information for**

**Yolk-Shelled Silver Nanowire@Amorphous Metal-Organic Framework for Controlled Drug Delivery and Light-Promoting Infected Wound Healing**

Yueyan Yang^1,2^, Xu Sun^1,2^, Shengyan Wang^3^, Zhe Tang^1,2^, Siyuan Luo^1,2^, Jianjun Shi^1,2^, Xiaolu Zhuo^3^, Jinjin Zhu^4^, Han Zhang^1,2*^, Xiangdong Kong^1,2*^

^1^ Institute for Smart Biomedical Materials, School of Materials Science & Engineering, Zhejiang Sci-Tech University, Hangzhou 310000, PR China

^2^ Zhejiang-Mauritius Joint Research Center for Biomaterials and Tissue Engineering, Zhejiang Sci-Tech University, Hangzhou 310018, PR China

^3^ School of Science Engineering, The Chinese University of Hong Kong, Shenzhen, Guangdong 518172, PR China

^4^ Department of Orthopaedic Surgery, Sir Run Run Shaw Hospital, Zhejiang University School of Medicine & Key Laboratory of Musculoskeletal System Degeneration and Regeneration Translational Research of Zhejiang, Hangzhou 310016, PR China

**^___________________________________________________________________________________________________________________^**

**^*^**Correspondence:

Han Zhang

[zhanghan@zstu.edu.cn](mailto:zhanghan@zstu.edu.cn)

Xiangdong Kong

[kongxd@zstu.edu.cn](mailto:kongxd@zstu.edu.cn)


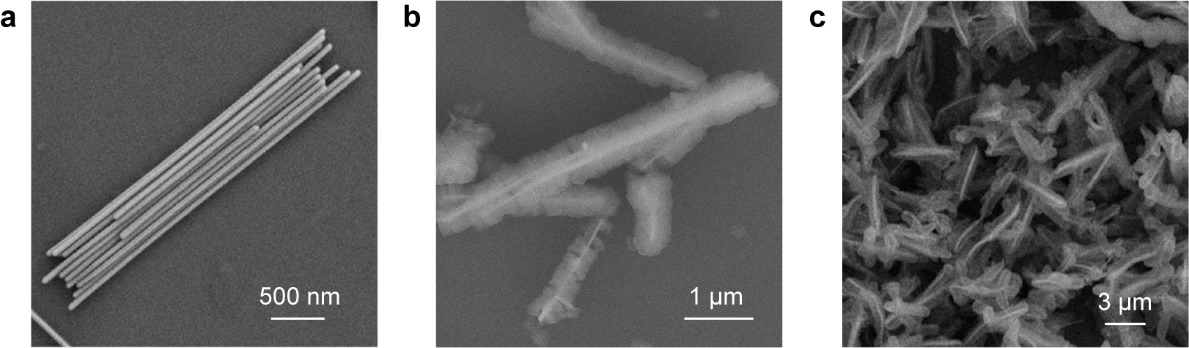


**Figure S1****.** SEM images of Ag NWs (a), Ag NWs@ZIF-67 (b) and Ag NWs@H-ZIF-67 (c).


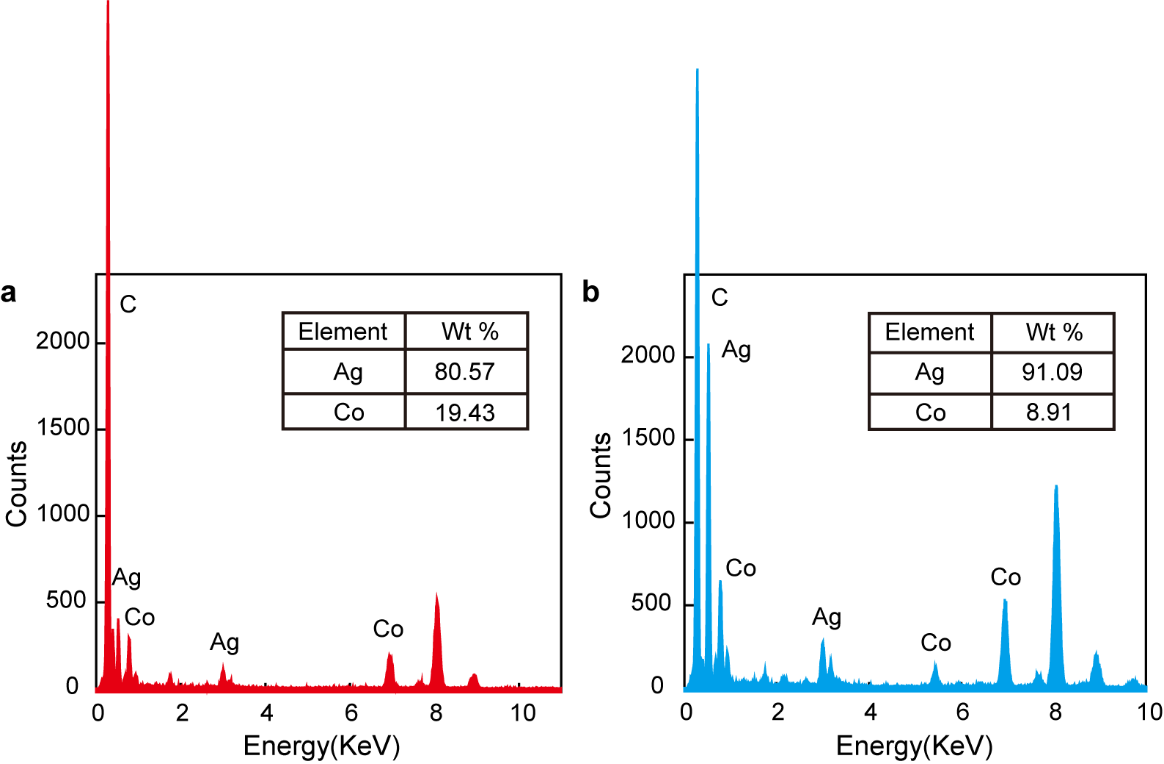


**Figure S2.** EDS spectra of Ag NWs@ZIF-67 (a) and Ag NWs@H-ZIF-67 (b).


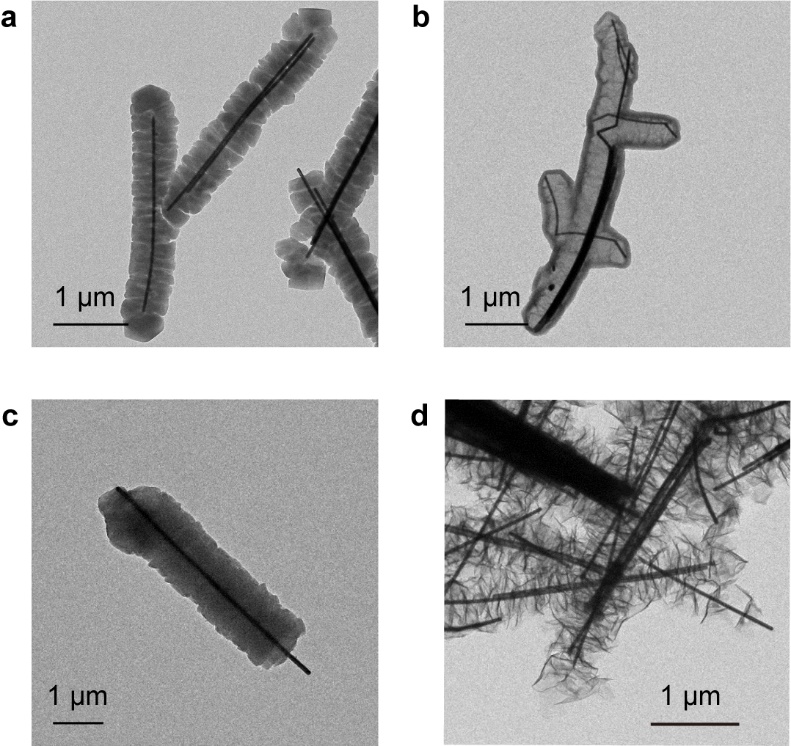


**Figure S3.** TEM images of Ag NWs@ZIF-67 (a) and Ag NWs@H-ZIF-67 (b) in methanol phase for 30 days, TEM images of Ag NWs@ZIF-67 (c) and Ag NWs@H-ZIF-67 (d) put in PBS for 12 h.


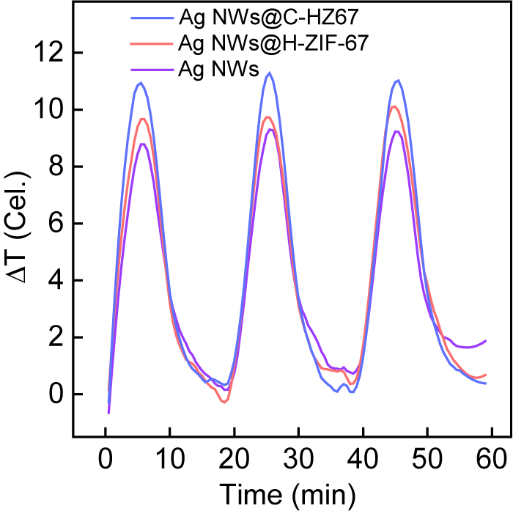


**Figure S4.** Photothermal-cooling cycles of Ag NWs, Ag NWs@H-ZIF-67 and Ag NWs@C-HZ67 (the optical density of Ag NWs =3) samples under the white light illumination by Xe lamp (0.5 W cm^‒^²).


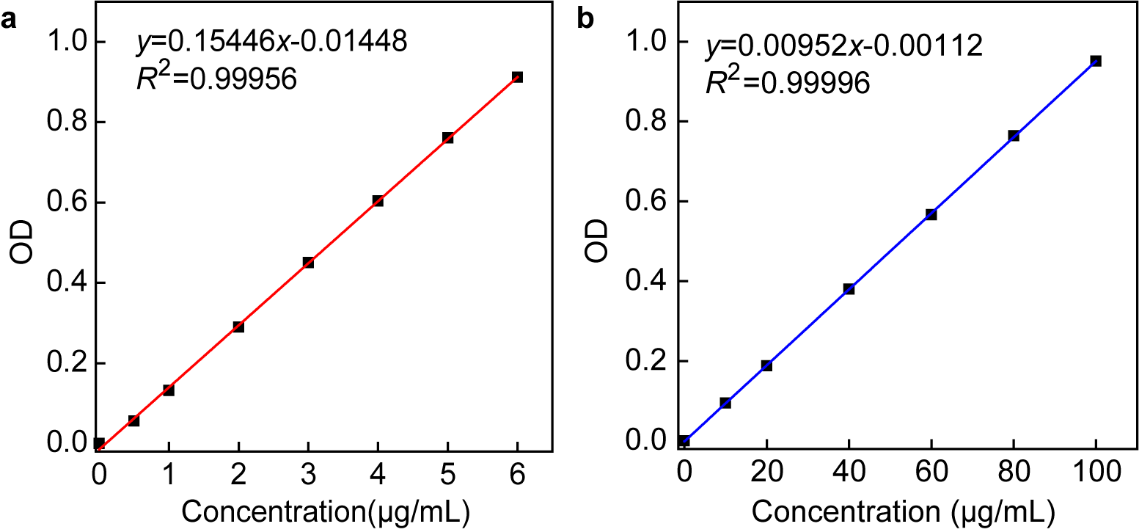


**Figure S5.** Linear relationship between the peak absorbance at 420 nm and the CCM concentration in methanol solution (a) and 0.5 v/v% PBS and Tween 20 solution (b).


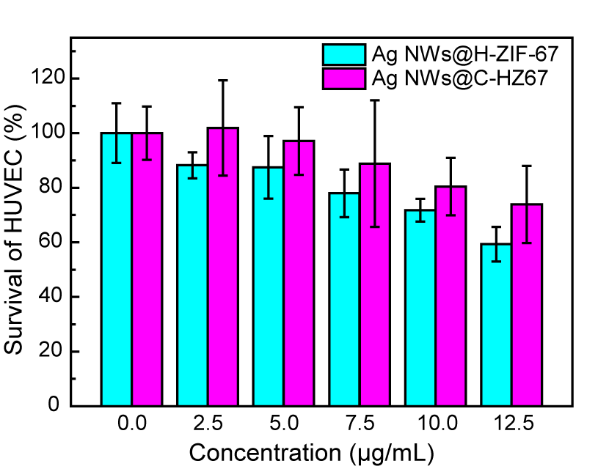


**Figure S6.** Cell viability of HUVEC cells after being incubated with Ag NWs@H-ZIF-67 and Ag NWs@C-HZ67 for 12 h.


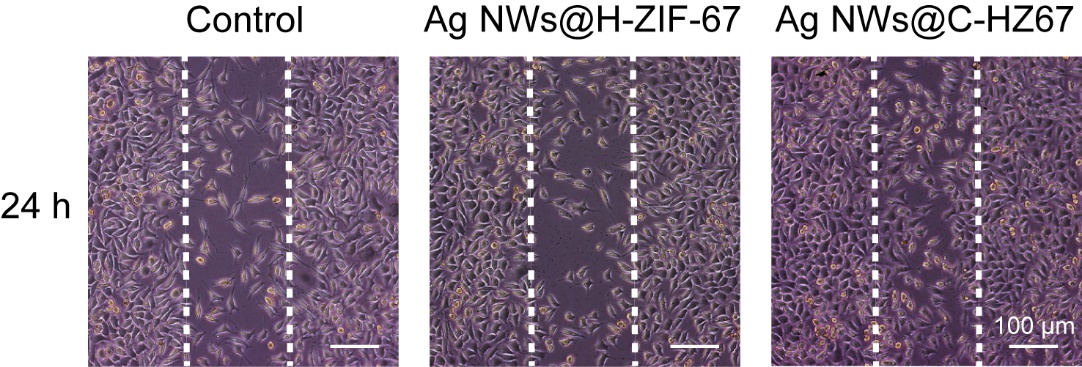


**Figure S7.** The migration of L929 cells in an *in vitro* scratch evaluation after 24 h of the incubation period. Scale bar: 100 µm.


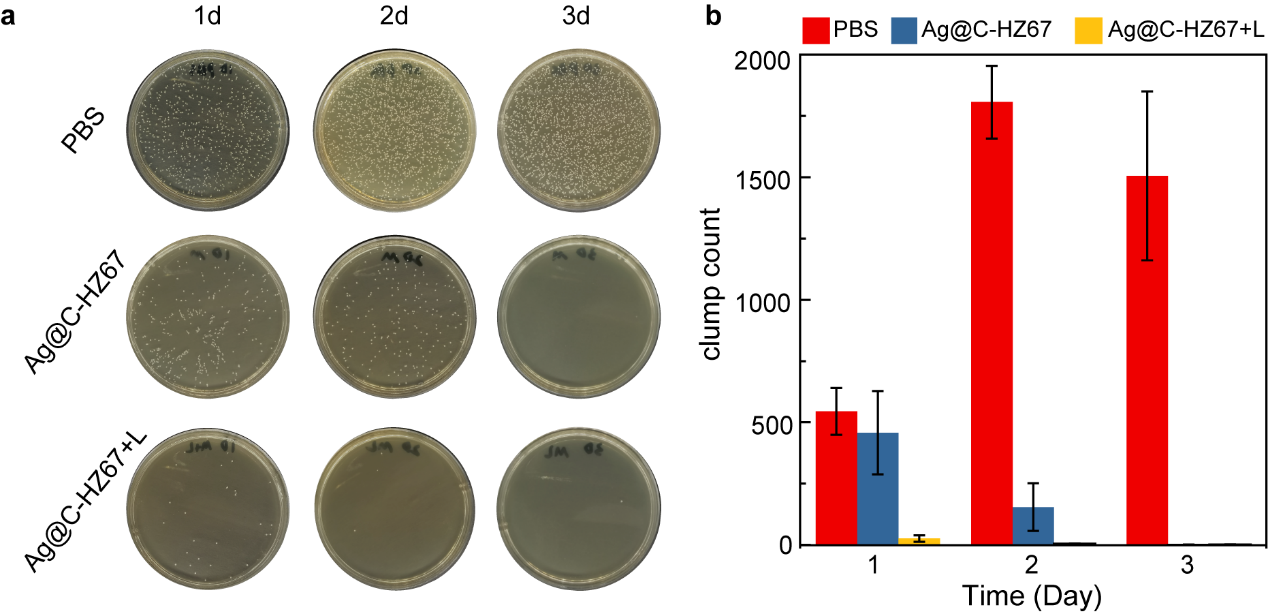


**Figure S8**. The bacterial contents in the wounds at days 1, 2 and 3 under the treatment of PBS, Ag NWs@C-HZ67, and Ag NWs@C-HZ67+L groups. Photographs of survival bacteria clones on agar plates (a). Wound surface colony statistics (b).


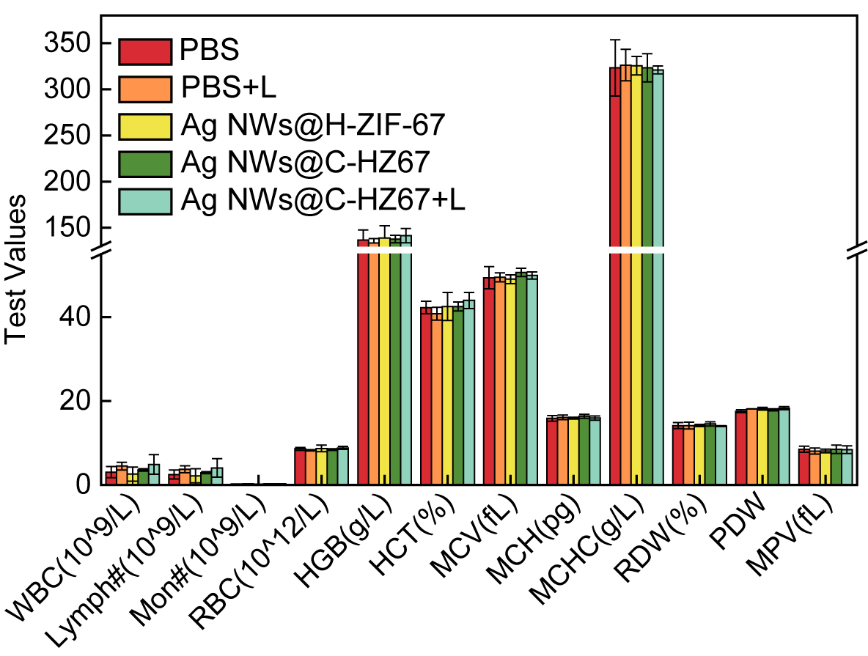


**Figure S9.** Blood routine of mice after various treatment.


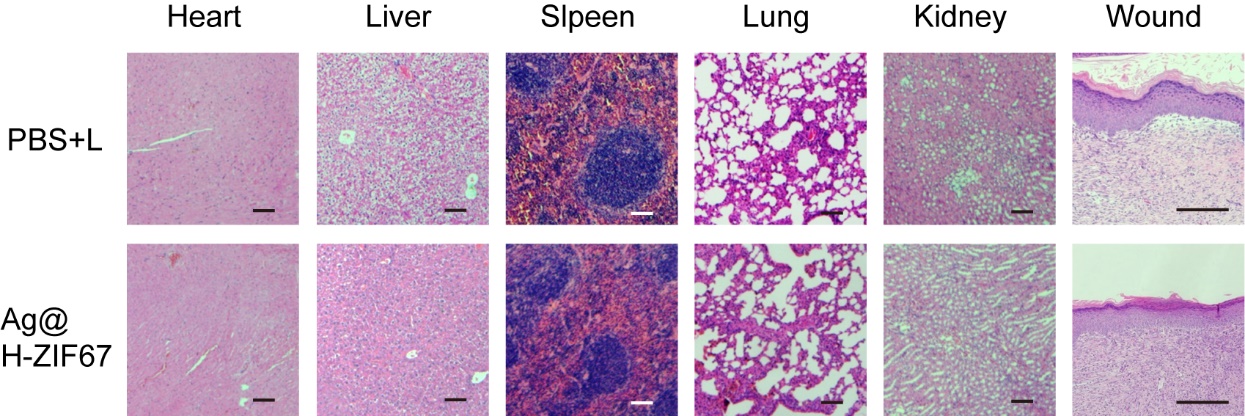


**Figure S10.** H&E-stained heart, liver, spleen, lung, kidney and wound photos taken at the 9th day after the mice treated with PBS+L, Ag NWs@H-ZIF-67, respectively. Scale bar: 200 μm.
